# Analytical Strategies to Analyze the Oxidation Products of Phytosterols, and Formulation-Based Approaches to Reduce Their Generation

**DOI:** 10.3390/pharmaceutics13020268

**Published:** 2021-02-16

**Authors:** George Gachumi, Asmita Poudel, Kishor M. Wasan, Anas El-Aneed

**Affiliations:** 1 Drug Discovery and Development Research Group, College of Pharmacy and Nutrition, University of Saskatchewan, Saskatoon, SK S7N 5E5, Canada; george.gachumi@usask.ca (G.G.); asp170@mail.usask.ca (A.P.); 2 iCo Therapeutics Inc., Vancouver, BC V6Z 2T3, Canada; Kishor.Wasan@ubc.ca; 3 Faculty of Medicine, University of British Columbia, Vancouver, BC V6T 1Z3, Canada; 4 Skymount Medical Group Inc., Calgary, AB T3C 0J8, Canada

**Keywords:** phytosterol oxidation products, analysis, GC-MS, LC-MS, delivery system, liposomes

## Abstract

Phytosterols are a class of lipid molecules present in plants that are structurally similar to cholesterol and have been widely utilized as cholesterol-lowering agents. However, the susceptibility of phytosterols to oxidation has led to concerns regarding their safety and tolerability. Phytosterol oxidation products (POPs) present in a variety of enriched and non-enriched foods can show pro-atherogenic and pro-inflammatory properties. Therefore, it is crucial to screen and analyze various phytosterol-containing products for the presence of POPs and ultimately design or modify phytosterols in such a way that prevents the generation of POPs and yet maintains their pharmacological activity. The main approaches for the analysis of POPs include the use of mass spectrometry (MS) linked to a suitable separation technique, notably gas chromatography (GC). However, liquid chromatography (LC)-MS has the potential to simplify the analysis due to the elimination of any derivatization step, usually required for GC-MS. To reduce the transformation of phytosterols to their oxidized counterparts, formulation strategies can theoretically be adopted, including the use of microemulsions, microcapsules, micelles, nanoparticles, and liposomes. In addition, co-formulation with antioxidants, such as tocopherols, may prove useful in substantially preventing POP generation. The main objectives of this review article are to evaluate the various analytical strategies that have been adopted for analyzing them. In addition, formulation approaches that can prevent the generation of these oxidation products are proposed.

## 1. Introduction

**Figure 1:**
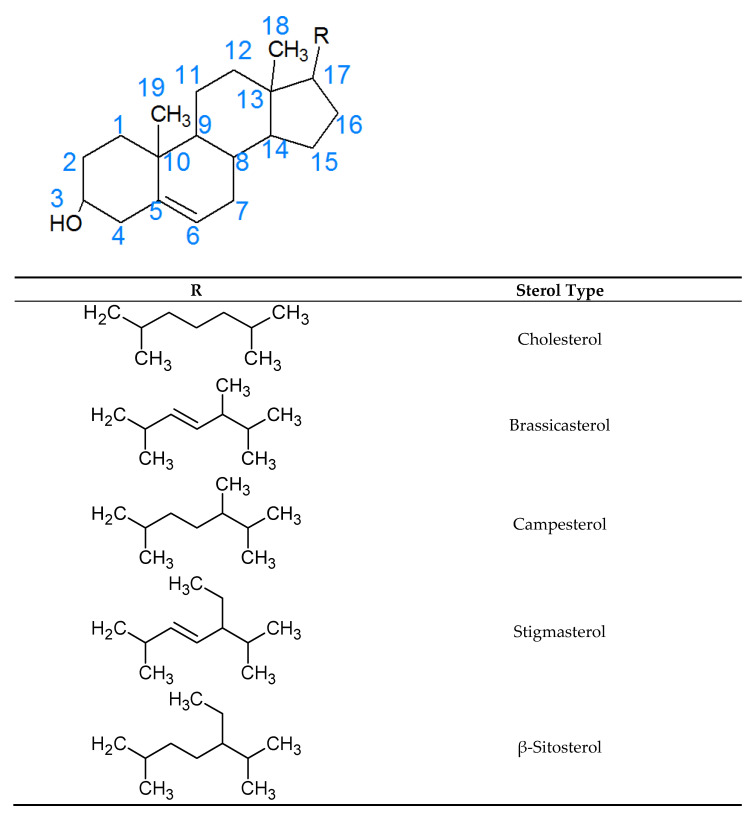
Nomenclature and structure of four abundant natural phytosterols (brassicasterol, campesterol, stigmasterol, and β-sitosterol), including cholesterol.

Phytosterols are secondary plant metabolites belonging to the triterpene family with a tetracyclic ring and a side chain linked to carbon 17 of the core structure (Figure 1). Brassicasterol, campesterol, stigmasterol, and β-sitosterol are the four most abundant phytosterols in plants (Figure 1). Natural phytosterols can be obtained from vegetable oils such as canola, sunflower, and soybean [1]. The United States Food and Drug Administration (USFDA), Health Canada, and the European Food Safety Authority (EFSA) have approved phytosterols as cholesterol-lowering agents [2,3,4]. This has led to the emergence of phytosterol-containing functional foods [5,6,7]. Various food products, including margarine [5], salad dressing [6], and orange juice [7], have been used in the development of phytosterol-enriched functional foods. Meta-analysis studies evaluating the efficacy of phytosterol-enriched foods in lowering low-density lipoprotein cholesterol (LDL-C) showed that daily intake of two grams of phytosterols lowered LDL-C by 8–10% over a 3-month period [8,9,10].

Since phytosterols are structurally similar to cholesterol (Figure 1), when they are consumed, they compete with cholesterol for absorption from the gastrointestinal track and reduce LDL-C levels by competing with cholesterol in the enterocytes [11]. Unlike cholesterol, of which up to 60% gets absorbed into the bloodstream, less than 2% of phytosterols undergo absorption [12]. However, in sitosterolemia, a rare genetic disorder, there is a significant absorption of phytosterols [13,14]. This absorption can lead to significant toxicity such as premature atherosclerosis [13,14]. Equally concerning is that phytosterol oxidation products (POPs) arising from the oxidation of phytosterols tend to undergo absorption, even in the case of individuals with normal genetic makeup [15,16].

**Figure 2:**
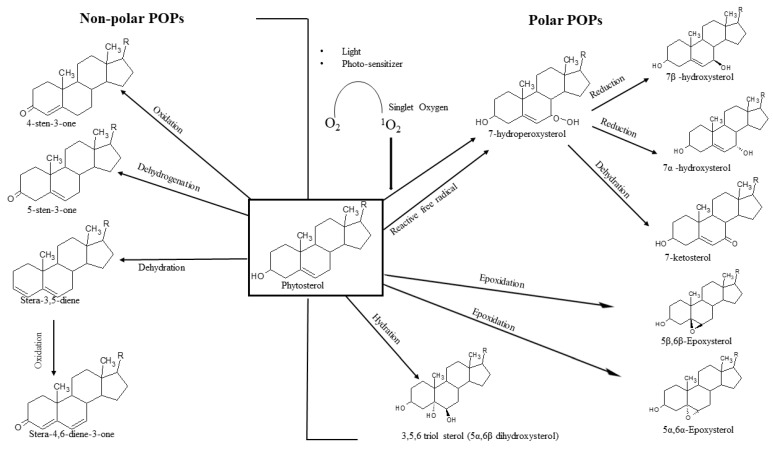
Auto-oxidation and photo-oxidation of phytosterols forming various polar and non-polar phytosterol oxidation products (POPs) [17,18].

Similar to cholesterol oxidation products, POPs are generated by auto-oxidation and photo-oxidation mechanisms (Figure 2) [17,18]. Primarily, POPs are formed by the oxidation of the steroid ring of phytosterol. As a result, POPs of diverse polarity are generated; however, the most abundant POPs are the polar ones such as 7-keto, 7-hydroxy, and 5, 6 epoxy [19,20]. Non-polar POP derivatives such as 3,5 dienes and 4,5 diene-3-one [17] are also formed, albeit at a lower extent. The mechanisms by which polar POPs are generated are shown in Figure 2. Apart from ring oxidation, the generation of POPs by oxidation of the side chain has also been reported [21,22]. However, the presence of side-chain-oxidized phytosterols in food products is low [23]. This is probably due to the fact that side chain oxidation of phytosterols is mediated by enzymatic reactions in vivo rather than by auto-oxidation [24].

Several studies have postulated the association between the absorption of POPs and the development of cardiovascular disease, primarily due to their possible pro-atherogenic effects that can possibly lead to atherosclerosis [24,25,26]. In addition, the high prevalence of POPs in phytosterol-enriched foods highlights the health risks of consuming these products on an ongoing basis [16,27]. It is, therefore, crucial to thoroughly explore POPs and investigate approaches that can minimize their generation to allow safe utilization of phytosterol-enriched products. Numerous published reviews demonstrated the negative health implications of POPs [16,22,28,29]. Furthermore, several reviews discussed their occurrence and the factors affecting their generation [16,22,27,28,30].

To the best of our knowledge, however, there is no detailed review that discusses analytical approaches to screen POPs alongside their occurrence and strategies to prevent their formation. Only few articles discussed the analysis briefly [16,28,31]. It is also highly important to explore the formulation approaches and strategies that can prevent the generation of POPs in phytosterol-enriched foods, something which appears to be currently lacking in the published literature. This article will review and discuss, in detail, the analytical approaches for screening and quantifying POPs and attempts to propose potential formulation strategies that can prevent their generation in phytosterol-enriched products.

## 2. Analysis of Phytosterol Oxidation Products

Analytical methods for the qualitative and quantitative analysis of phytosterols and cholesterol oxidation products (COPs) are prevalent [32,33,34,35,36] and may serve as a starting point for the development of targeted POP analytical methods, due to their structural similarities. However, the analysis of phytosterols and COPs will not be discussed in detail in this review, which is focusing on the analysis of POPs.

POP analyses are challenging due to limitations in the availability of reference standards. Most of the reported methods have quantified POPs tentatively, using COPs as a reference standard [17,37,38]. However, advancements in the purification or synthesis of POPs have made it possible to generate suitable standards, ensuring accurate quantification. For example, both deuterated and non-deuterated reference POPs with the desired stereochemistry have been chemically synthesized [39,40,41]. Reference POPs via purification from thermally oxidized phytosterols by reversed- or normal-phase liquid chromatography have been reported, although chemical synthesis is sometimes incorporated due to the inability of one approach to prepare all desired POPs [19,42].

As shown in Figure 2, major POPs arise from oxidation reactions on the steroidal ring. Therefore, the most widely quantified derivatives are hydroxy, epoxy, and keto (Figure 2) [20,43]. Generally, POPs are contained in the bulk lipids of the sample matrix and are part of the unsaponifiable matter. However, they are also present in non-lipid matrices such as beverages with added phytosterols, e.g., fruit juices. POPs are present in relatively low amounts compared to un-oxidized phytosterols, and for accurate identification and quantification, efficient extraction and purification steps are necessary.

### 2.1. Extraction and Enrichment of POPs

Sample preparation is an indispensable process for both qualitative and quantitative analysis of POPs. Efficient sample preparation enhances the sensitivity of an analytical method due to the minimization or elimination of extraneous components that contribute to interferences. Such interferences pose challenges during chromatographic separation and may lead to a compromise in the selectivity and sensitivity of an analytical approach.

As mentioned earlier, POPs are generally part of the bulk lipids and part of the unsaponifiable matter in the case of oils. Sample pretreatment, therefore, includes an initial extraction of total lipids followed by saponification/transesterification or can be a direct saponification/transesterification. Liquid–liquid extraction of POPs is then performed on the saponified or transesterified samples (Table 1). In cases where the matrix is non-lipid, such as in beverages, saponification and transesterification steps are not necessary and can be omitted [44,45].

The initial step for total lipids extraction is to reduce the matrix complexity and enhance the recovery of POPs. The commonly used extraction procedures are the Folch [46] and the Bligh and Dyer [47] methods that use a mixture of chloroform and methanol. However, due to safety, health, and environmental concerns, changes are adopted to replace hazardous solvents. Despite changes in the extraction processes, some alternative approaches that can yield comparable extraction efficiency are still toxic due to the use of hexane, for example. Comparatively, hexane is more non-polar than chloroform, and using it for extraction would result in a different profile. In fact, the Folch method (chloroform/methanol 2:1) was found to be less selective for lipids in egg-containing foods as non-lipid fractions were also extracted. However, a mixture of hexane/isopropanol (3:2) provided the desired selectivity [48].

Saponification of lipids is more common compared to transesterification, as shown in Table 1. The former eliminates fatty acids and any other saponifiable matter, while the latter leads to the formation of fatty acid methyl esters (FAMEs) that comprise the bulk of the extract. Although further tests with other food matrices are needed, transesterification was reported to give better recovery of POPs in rapeseed oil (98.9%) compared to cold saponification (66.3%) [49]. For the extraction of POPs after saponification or transesterification, dichloromethane seems to be the solvent of choice, although chloroform and diethyl ether have also been used (Table 1). It is likely that dichloromethane, a moderately polar solvent, has high selectivity for POPs and its low boiling point enhances its removal via evaporation. To increase the purity of the extracted POPs, purification and enrichment techniques such as solid phase extraction (SPE), thin layer chromatography (TLC), and preparative liquid chromatography are employed. These techniques are described in the following sections.

#### 2.1.1. Solid Phase Extraction (SPE)

Solid phase extraction has been applied both in the preparation of POP reference standards as well as for the purification of POPs from a sample matrix after liquid–liquid extraction. The use of silica as an adsorbent is widely utilized. For example, using silica cartridges, POPs were purified from serum samples [39,40]. The procedure involved a conditioning process with n-hexane, a washing step with 0.5% 2-propanol in n-hexane, and elution with 30% 2-propanol in n-hexane. In another study, POPs were purified from serum where cyclohexane was employed for conditioning, a washing step with 0.5% ethyl acetate in cyclohexane, and elution with ethyl acetate [41]. In effect, the extraction solvent mixture should possess medium polarity, allowing for efficient extraction.

Similarly, POPs were purified from vegetable oils; however, diethyl ether was used for washing and acetone for elution [43,49,53]. Whereas the discussed procedures employ a single-step SPE, two-step SPE has been reported for the enrichment of POPs from vegetable oils and food products [51,52,54]. Using a silica cartridge, POPs were enriched from deep-fried potato chips by performing the procedure twice [54]. The two-step SPE allowed the complete removal of any unoxidized sterols from the unsaponifiable matter. Similarly, using an amine SPE, POPs were enriched from transesterified samples of vegetable oils, vegetable fat spreads, and breaded shrimp by performing the procedure twice [51,52]. Two-step SPE was necessary to eliminate fatty acid methyl esters (FAMEs) that co-elute with POPs during single-step SPE [51]. Although silica is preferred in comparison to amine adsorbent, the choice of the solvent system may be the key to a successful purification. In fact, with an optimized solvent system, a single-step SPE using silica has been reported for the purification of POPs from a transesterified sample [49]. The column was conditioned with hexane, followed by a sequential wash as follows: low-polarity lipids (non-POPs) were washed with hexane/diethyl ether (9:1 *v*/*v*), polar lipids (non-POPs) with hexane/diethyl ether (1:1 *v*/*v*), and, finally, POPs were eluted using acetone. This procedure was successful as there were no FAMEs or unoxidized sterols detected in the enriched fraction. Similar results were obtained with the same SPE procedure when an optimized solvent system was adopted for the purification of POPs from saponified edible oils [55].

#### 2.1.2. Thin Layer Chromatography (TLC) and Preparative Liquid Chromatography

Compared to SPE, which is considered a rapid procedure, TLC and preparative liquid chromatography are tedious and time-consuming [49]. Nonetheless, they have been applied for the enrichment and purification of POPs. These techniques, however, are ideal for obtaining a purified single compound as compared to SPE, which yields a purified mixture belonging to a class of compounds. In fact, some studies have combined both SPE and TLC/liquid chromatography to isolate specific POP derivatives. For example, POP reference standards were prepared by both thermal oxidation and chemical synthesis in order to determine their levels in enriched and non-enriched spreads [42,50]. Both chemically synthesized and thermally oxidized POPs were purified using SPE and then subjected to a preparative TLC. While keto, epoxy, and hydroxy derivatives were effectively isolated from thermally oxidized phytosterols, the triol derivatives were not and were chemically synthesized. These studies identified the presence of non-oxidized phytosterols during TLC, highlighting the inefficiency of SPE to completely eliminate non-oxidized phytosterols [42,50]. Such a shortcoming has been addressed via an improved SPE that allows complete separation of POPs from non-oxidized sterols by sequential washes with solvents possessing varying polarities [49]. In addition, TLC, with an optimized solvent system, was employed in the identification of side-chain-oxidized products of stigmasterol, sitosterol, and campesterol. The desired separation was achieved for hydroxy derivatives at carbons 24 and 25 of stigmasterol, campesterol, and sitosterol [21,56].

Another approach, though not widely employed, is the application of preparative liquid chromatography. Normal-phase liquid chromatography (NPLC) was utilized in purifying POPs in cooked and baked products [38]. The employed eluent was hexane/isopropanol; however, gradient elution was used and the whole process required 50 min. Scholz et al. [19] used a similar elution profile for the preparation of POP reference standards from thermally oxidized phytosterols with a total run time of 30 min. However, POP enrichment and analysis were performed using on-line liquid chromatography. Unlike the dual step where liquid chromatography is performed separately followed by analysis, on-line LC-GC was used. The LC part allowed the enrichment of POPs from a transesterified sample via chromatographic separation, and the fractions were injected into GC for identification and quantification.

After various sample preparation steps, namely extraction, purification, and enrichment, the commonly utilized analytical approaches for the qualitative and quantitative analysis of POPs are gas and liquid chromatography. These chromatographic separation techniques are used in conjunction with mass spectrometric or flame ionization detection methods. It should be noted that a harmonization of analytical methods for the analysis of cholesterol and non-cholesterol sterols has been conducted [57]. However, discrepancies were found among the results submitted by the participating laboratories, indicating the need for additional efforts to ensure the reproducibility of the results. A similar approach for analytical method harmonization for the analysis of POPs is highly recommended to ensure the quality of the reported results.

### 2.2. Gas Chromatography

Gas chromatography is ideal for the analysis of non-polar compounds; however, most POPs possess varying polarities. Therefore, derivatization is required to enhance the volatility of POPs. As indicated, qualitative and quantitative analyses have been performed using gas chromatography linked to either a flame ionization detector (GC-FID) or mass spectrometer (GC-MS). While the former was mainly successfully used for quantification [19], the latter is adapted for both identification and quantitation [42,58]. Authentic standards are required for analysis using GC-FID, while identification can be carried out in GC-MS without the use of reference standards due to the presence of comprehensive mass spectrometric libraries.

POPs have been commonly quantified using COPs as reference standards with the assumption that the response factors of POPs are similar to those of COPs [38]. In addition, the use of 5α-cholestane, 19-hydroxycholesterol, or the deuterated form of hydroxycholesterol as internal standards is common [38,42,51,54,59]. Recently, the use of synthesized POPs and their deuterated form as internal standards has also been reported [39,40].

GC-MS was employed in monitoring the formation of sterol oxides of both saturated and unsaturated phytosterols in rapeseed oil and tripalmitin matrix [53,58]. A full scan mode (*m/z* 100–600) was used for identification and single ion monitoring mode (SIM) for quantification. Sample enrichment was achieved with SPE, followed by derivatization and subsequent analysis of the silylated derivatives. Hydroxy, epoxy, and keto derivatives were identified for stigmasterol, while only hydroxy derivatives were reported for sitostanol, highlighting the impact of the structure on the produced POPs [58]. One oxidation product of sitostanol was reported to be in relative high abundance in thermally oxidized standards, rapeseed oil, and tripalmitin matrix, but it was not identified [53,58]. It was, however, quantified by employing the unidentified compound in thermally oxidized phytosterols as a reference standard. Based on its GC elution, it was hypothesized to be a hydroxy or keto derivative of sitostanol [53,58]. The presence of such unidentified compounds highlights the need for authentic reference standards for all possible POPs that can potentially form so that proper identification is achieved.

POPs have been monitored during frying of various food products in vegetable oils [52,60,61]. In French fries, hydroxy, epoxy, keto, and triol derivatives of campesterol, stigmasterol, and sitosterol were quantified using GC-FID, while GC-MS was utilized for identification. The total content of POPs ranged from 16.8 to 147.6 µg/g and was dependent on the heating time and frequency [60]. However, brassicasterol oxidation products were not determined due to difficulties in their identification. Although no explanation was provided, it can be speculated that either a loss during sample preparation or a lack of authentic standards was the reason for not reporting brassicasterol oxidation products. In meat, POPs were undetectable before frying; however, their concentration range was 0.1–1.6 µg/g after frying as a result of phytosterol oxidation in oil during the cooking process [61]. Full scan MS was employed and only epoxy and keto derivatives of campesterol and sitosterol were identified. In both the French fries and the meat, rapeseed oil was used, and it can be hypothesized that the variation in the composition and quantity of the identified POPs is due to variation in cooking methods and duration. Similarly, by employing full scan MS (*m/z* 100–600), stigmasterol triol and hydroxy derivatives of sitosterol/stigmasterol were identified in cooking oil (soy bean) but not detected in the baked or fried breaded shrimp [52]. Although POPs were expected in the baked and fried breaded shrimp, they were not detected, and the authors suggested that any POPs produced may have been below the detection limits of the instrument [52].

Improved sensitivity in GC analysis has been reported due to improvements in sample preparation. For example, in the analysis of POPs in cooked and baked foods using GC-SIM-MS, the extraction process was optimized to match the sample matrix [38]. This resulted in improved sensitivity and reproducibility, and the POP detection limit was 0.02 mg/kg. Hu et al. [55] developed and validated a GC-SIM-MS method for determining POPs in edible oils. The derivatization was optimized and the derivatization agent that showed the highest response in GC-MS was adopted for quantification. An enhancement in detection sensitivity of POPs was observed, and the detection limits were less than 2.99 ng/mL for hydroxy, triol, and keto derivatives while it was in the range of 14.6–36.3 ng/mL for epoxide derivatives. These values were, however, low enough to allow for the monitoring of target compounds in experimental oil samples. In another study, a GC-SIM-MS method was developed and validated for the analysis of POPs in human serum. Improved detection limits were reported and ranged from 1.23 to 4.14 ng/mL for hydroxy, triol, keto, and epoxide derivatives [37]. It should be noted that the level of method validation in the case of sterols and oxidation products varies based on the intended application. While full validation is expected for human clinical trials, a fit-for-purpose approach can be sufficient to answer the research question in the case of a food or vegetable oil analysis. Both validated and non-validated methods are discussed in this paper.

To improve the separation of POPs, the use of two different columns to separate various POPs has been reported using GC-FID and GC-MS in phytosterol-enriched foods. Two different columns, DB-1 (non-polar) and DB-5 (slightly polar), were compared for their chromatographic separation, showing that approximately 0.08% (68 µg/g) of phytosterols were oxidized [42]. Some POPs showed good separation on one column, while they were unresolved on the other. For example, campestanetriol and α-epoxysitosterol were resolved in DB-1 and unresolved in DB-5, while 6β-hydroxysitosterol and 6-ketocampestanol were resolved in DB-5 and not in DB-1. The data obtained from the two columns were compared and used for identification and quantification [42]. Although phytosterols oxidized on the side chain were expected, they were not detected, and it was hypothesized that the employed SPE may have been responsible for their elimination [42]. In contrast, GC-FID with two columns—DB35-MS (moderately polar) and DBS MS (non-polar)—connected in series was used for the separation and analysis of various POPs in vegetable oils and spreads enriched with phytosterol esters [51]. The identified and quantified POPs were hydroxy, keto, epoxide, and triol derivatives of campesterol, stigmasterol, and sitosterol, including side chain oxidation products (24-hydroxy).

Although the above-discussed GC methods utilized COPs as reference standards, few studies have been reported where POP reference standards were used. Through the synthesis of both POP reference standards and deuterated internal standards, POPs were analyzed using GC-SIM-MS in human serum [41]. Using POP standards instead of COPs resulted in improved sensitivity in the determination of POPs in serum, with detection limits in the range of 7–243 pg/mL [39,40]. It can be argued that the use of authentic POP reference standards and internal standards contributes to the sensitivity and accuracy of the method, as the calibration curves reflect the true response of the individual analyte.

#### GC-MS/MS

To enhance the selectivity of POPs during analysis, tandem mass spectrometry (MS/MS) can be used. Due to the lack of commercially authentic reference standards of POPs, GC-MS/MS offers a reliable method of identification. Reference POPs obtained via thermal oxidation can be easily identified by analyzing their MS/MS fragmentation pattern. In addition, MS/MS data can be extended for the identification of other unidentified POPs within a sample due to structural similarities. In fact, a generalized MS/MS fingerprint was successfully used for the identification of structurally related sterols [62].

In many reported studies, GC-MS/MS was utilized for the identification of POPs in phytosterol-enriched foods, while quantification was performed using GC-FID [63,64,65,66]. In these studies, calibration curves were generated using COP reference standards. Sitosterol is the most abundant phytosterol, and its oxidation products were found as the dominant POPs followed by those of campesterol. While only sitosterol oxidation products were quantified in fruit- and milk-based beverages, no additional information was provided regarding the identification of other POPs [64,66]. However, quantification of both sitosterol and campesterol oxidation products in fruit juices, milk-based beverages, and commercial ingredients containing phytosterols was reported [63,65]. In all these studies, the sources of phytosterols were tallow oil and/or vegetable oils such as rapeseed, soybean, sunflower, and corn. It is unclear why oxidation products of stigmasterol and brassicasterol (from rapeseed source), though expected, were not identified and measured in the tested phytosterol-enriched products.

GC-MS/MS has also been scarcely utilized for the quantitative analysis of POPs. Both hydroxyl and keto forms of sitosterol and campesterol oxidation products were identified and quantified in human serum [67,68]. The concentrations of POPs were found to increase after consuming phytosterol-enriched food. To enhance the separation power of GC for phytosterols, some studies utilized two-dimensional GC (GCxGC) for the analysis of phytosterols. GCxGC requires the use of columns with different separation mechanisms. Utilizing GCxGC coupled with time-of-flight mass spectrometry (ToF-MS), POPs were analyzed in human plasma [20]. Improved resolution was achieved as co-elution was minimized [20]. In addition, 18 additional POPs including side-chain-oxidized brassicasterol and stigmasterol at the unsaturated C22–C23 position were identified [20].

Although GC-MS and GC-FID are widely applied for the analysis of POPs, the application of liquid chromatography (LC) is promising due to its simplicity in comparison to GC, as no derivatization is needed.

### 2.3. Liquid Chromatography

Compared to GC, liquid chromatography (LC) is operated at mild temperatures. In addition, tedious derivatization procedures can be avoided, hence simplifying the analytical approach. Qualitative and quantitative analyses of POPs can be performed in either normal- or reversed-phase chromatography. Saynajoki et al. utilized normal-phase liquid chromatography with two detectors, ultraviolet (UV) and fluorescence, to analyze stigmasterol oxidation products [69]. These detectors, however, cannot be selective for co-eluting sterol oxidation products. Thus, mass spectrometry has been the detector of choice for POP analysis when LC is used.

#### Liquid Chromatography–Mass Spectrometry

LC-MS can be performed using various atmospheric pressure ionization techniques, such as electrospray ionization (ESI), atmospheric pressure chemical ionization (APCI), and atmospheric pressure photoionization (APPI). A reversed-phase LC-MS method was developed using ESI for the characterization of a variety of synthetic stigmasterol oxidation products, and nuclear magnetic resonance (NMR) was also used to complement MS results [70]. However, ESI is not ideal for ionizing hydrophobic compounds as it shows lower ionization efficiency [71].

Alternatively, APCI and APPI can be utilized for efficient ionization of POPs. Using APCI, POP fragmentation was studied, and a normal-phase LC-MS method was developed [72]. All of the non-polar POPs and 7-ketosterol ionized as [M + H]^+^, whereas relatively unstable polar POPs such as 7-hydroxysterol, 5,6-epoxysterol, and 5,6-dihydroxysterol were found to undergo in-source fragmentation, resulting in the formation of [M + H − H_2_O]^+^ and [M + H − 2H_2_O]^+^ species [17]. As expected, β-sitosterol, campesterol, stigmasterol, and brassicasterol oxidation products were successfully analyzed without the need for derivatization [72]. In fact, both APCI and APPI showed similar ionization behaviors for POPs and have been used in the analysis of various phytosterol oxidation products in normal-phase LC-MS [17,73]. However, one of the drawbacks of normal-phase LC methods is the long analysis time, reaching 30 min [17,73]. To shorten the LC-MS run time, reversed-phase chromatography can be used [70]. Unfortunately, to the best of our knowledge, none of the published APCI/APPI-LC-MS methods for POPs have utilized reversed-phase LC.

**Figure 3:**
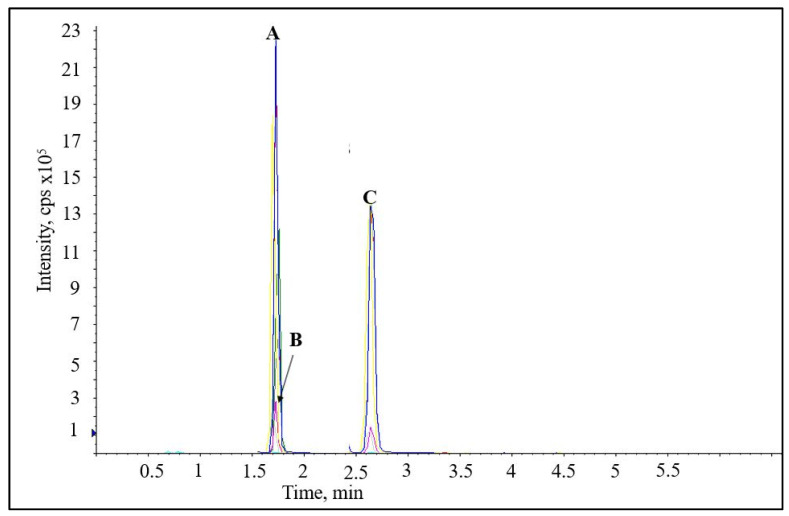
Liquid chromatography–tandem mass spectrometry (LC-MS/MS) chromatographic separation of 7-keto (**A**), 7-hydroxy (**B**), and 5, 6 epoxy (**C**) cholesterol using reverse-phase chromatography.

Nevertheless, reversed-phase chromatography has been adopted for quantifying cholesterol oxidation products (COPs) in food products and human serum [32,33]. An LC-APCI-MS method using a C18 analytical column was able to quantify polar COPs (25-hydroxy, 3,5,6 triol, 7-hydroxy, 7-keto, 5,6 epoxy) present in processed food with a run time of ten minutes [32]. A similar method quantified five different polar COPs in human serum in merely five minutes [33]. Both studies can be extrapolated to POPs for developing a short analytical method. In fact, our group is currently in the final stages of validating a novel LC-MS analytical method for the determination of three commonly encountered POPs and will be communicated upon completion. A representative chromatogram is shown in Figure 3.

## 3. Possible Formulation Strategies to Prevent the Formation of POPs

Two major formulation strategies can be adopted to prevent or minimize the generation of POPs, namely (i) the formulation of phytosterols in a suitable delivery system [74,75,76,77] and (ii) the addition of antioxidants to the phytosterol formulation [78,79].

### 3.1. Formulation of Phytosterols in Suitable Delivery Systems

Formulation of phytosterols into delivery vehicles such as microemulsion, micelles, microcapsule, microparticle, nanoparticles, and liposomes can prevent or at least minimize the formation of POPs. In fact, many delivery vehicles have already been explored to deliver phytosterols orally in order to increase their therapeutic efficacy (Table 2). For instance, phytosterols incorporated in lecithin micelles were able to reduce serum LDL-cholesterol by up to 14% [80]. Similarly, water-dispersible phytosterol formulation, composed of fatty acids and polysorbate, reduced LDL-cholesterol by up to 12% [81]. Liposomes have also been explored to formulate and test the efficacy of semi-synthetic phytostanol, a saturated derivative of phytosterols [74]. Even though most of these delivery vehicles enhanced phytosterols’ therapeutic efficacy, their ability to minimize the formation of POPs is still unknown (Table 2). However, the literature has clearly depicted the promising capabilities of liposomes, solid nanoparticles, micelles, and microemulsion in preventing the oxidation of various thermosensitive bioactives, such as resveratrol, β-carotene, vitamin D, and curcumin [82,83,84,85]. Despite their potential, it should be noted that no current studies have systematically evaluated the use of the above delivery systems in preventing the oxidation of phytosterols. However, mounting evidence suggests their usefulness in preventing oxidation of similar compounds. The brief discussions below are intended as a guide to the possibility of extending their use towards phytosterols.

Liposomes are colloidal systems made up of phospholipids, arranged as a lipid bilayer and aqueous core, and, thus, can entrap or encapsulate both hydrophilic and lipophilic compounds [89]. Efficient entrapment prevents bioactives from direct exposure to reactive free radicals and, in turn, prevents oxidation. In addition, liposomes made up of partially unsaturated phospholipids possess antioxidant properties, increasing the stability of the entrapped bioactives [90]. Thermosensitive bioactives such as resveratrol and β-carotene showed improved stability profiles upon liposomal entrapment [82,91]. These studies provide strong evidence for liposomes to prevent the oxidation of thermosensitive compounds. Thus, phytosterol oxidation will most likely be prevented by liposomal entrapment. Microemulsions are another delivery system that is gaining popularity in formulating or encapsulating bioactive components [92,93]. Similar to liposomes, microemulsions composed of oil, water, and surfactants (i.e., a colloidal dispersion) [83] can prevent oxidation of bioactive components [92,93]. Unfortunately, one of the major drawbacks of microemulsions is the requirement of large quantities of emulsifiers and surfactants that can be deleterious to human health [94].

In addition to liposomes and microemulsions, there is a trend in the utilization of other delivery vehicles such as micelles and nanoparticles. Both micelles and nanoparticles encapsulate or enclose bioactive compounds, thus preventing their direct exposure to reactive free radicals. For instance, micelles made up of β-casein and polyethylene glycol derivative were able to enhance the oxidative stability of vitamin D and polyphenols in comparison to free compounds [84,85]. In sum, there is strong evidence that supports the notion that liposomes, microemulsions, micelles, and nanoparticles can prevent the generation of POPs.

However, before the utilization of these vehicles as delivery agents, it is crucial to assess their ability to prevent phytosterols’ oxidation by conducting active ingredient stability studies. Furthermore, it is important to note that the majority of marketed phytosterol products have been designed as functional foods, while only few products are intended as supplements [95]. Thus, it is crucial for phytosterol delivery vehicles to fit both these applications. All the above-discussed delivery systems can be efficiently utilized for designing supplements. However, only micelles, microemulsions, and liposomes are advisable for functional foods due to their food-compatible components such as β-casein and lecithin. Nanoparticles (particularly inorganic nanoparticles), on the other hand, do not seem to be compatible with food components [96,97].

### 3.2. The Addition of Antioxidants to the Formulation

There is a trend of co-administration of antioxidants such as tocopherols, phenolic acids, butylated hydroxyl anisole, and butylated hydroxyl toluene along with bioactive compounds in order to prevent harmful oxidation [78,79]. The co-administration of tocopherols and polyphenols along with β-sitosterol significantly minimized the generation of POPs in comparison to the ones without antioxidants [78,79]. Both natural and synthetic antioxidants of diverse classes have been utilized for this purpose [78,79]. However, there is a variation in the antioxidant activity of these compounds as it depends upon two major factors—(i) the compound’s class and (ii) the compound’s source (natural or synthetic). For instance, tocopherols showed higher antioxidant activity to protect β-sitosterol and campesterol in comparison to butylated hydroxyl toluene (BHT) [78,98]. In one study, tested phytosterols were heated at 180 °C for 4 h [78], while in the other, phytosterols were heated at 180 °C for 2 h [98]. However, in the case of antioxidant activity between synthetic and natural tocopherols, the literature shows conflicting results. A study by Kmiecik et al. showed higher activity of synthetic tocopherols in comparison to natural ones in preventing oxidation of β-sitosterol and campesterol [78]. On the other hand, in vivo studies conducted in a piglet animal model showed higher antioxidant activity of natural tocopherols in comparison to synthetic ones [99]. Therefore, more studies are needed to obtain a conclusive recommendation for the use of antioxidants.

In sum, suitable formulation strategies and the addition of antioxidants are two different strategies that can be adopted to prevent the formation of POPs. It is even ideal to adopt both strategies together to impart higher oxidative stability. In fact, recently, in our lab, we designed a novel liposomal phytosterol formulation that contains tocopherols [100]. The ability of this novel formulation to prevent POP generation is currently being investigated.

## 4. Conclusions

Despite phytosterols’ promising effects as cholesterol-lowering agents, there is concern regarding their effective utilization in food due to their high susceptibility to oxidation. To assess the presence of potentially harmful POPs in food products, several analytical platforms such as GC-MS and LC-MS have been utilized. However, analytical challenges in the screening and quantification of POPs need to be addressed. The main challenges are the lack of authentic standards, the requirement of derivatization in GC-MS, and the longer analytical run time in LC-MS. In addition, both extraction and purification methods play a key role in the success of an analytical strategy to analyze POPs. The extraction, purification, and discussed analytical approaches have shown varying results, and the suitability of each analytical strategy should be evaluated on a case-by-case basis. There are also observed variations in the identified POPs from thermally oxidized phytosterols using GC. This is probably due to the matrix in which POPs are formed. In addition, the content of POPs in some food products might have been underestimated due to the lower sensitivity of existing analytical techniques. Further research is needed, such as synthesizing more authentic standards, developing faster methods, and opting for highly sensitive analytical instruments.

Various formulation strategies should also be explored to prevent oxidation of thermosensitive phytosterols. Designing phytosterols in an optimum formulation followed by screening for the presence of POPs at different storage conditions is advised before marketing phytosterol-enriched food products. This will ultimately lead to the safe utilization of phytosterols as food additives and nutraceuticals.

## Figures and Tables

**Table 1:** Sample pretreatment procedures and organic solvents used during liquid–liquid extraction of POPs.

Sample Matrix	Pretreatment Step	Extraction Solvent	References
Vegetable fat spread	Transesterification	Chloroform	[19]
Cooked/baked products	Lipid extraction/Saponification	Various ^b^/Dichloromethane	[38]
Serum	Saponification	Dichloromethane	[39,40,41]
Vegetable fat spread	Lipid extraction/Saponification	Dichloromethane/methanol (2:1) ^a^/Dichloromethane	[42]
Beverages	NA	Chloroform/methanol (2:1) ^a^	[44]
Hazelnut oil	Lipid extraction/Transesterification	Hexane:isopropanol (3:2) ^a^/Chloroform	[49]
Rapeseed oil	Saponification	Dichloromethane	[49]
Vegetable fat spread	Lipid extraction/Saponification	Hexane:isopropanol (3:2) ^a^/Diethyl ether	[50]
Maize oil	Transesterification	Dichloromethane	[51]
Breaded shrimp	Saponification	Dichloromethane	[52]

^a^ Solvent system for total lipid extraction; ^b^ solvent system was different for each sample: Fish—chloroform/methanol (1:2); Meat—chloroform/methanol (2:1); Vegetables and potatoes—Hexane/isopropanol (3:2); Baked products and eggs—petroleum ether.

**Table 2:** Various formulation approaches utilized for delivering phytosterols.

Phytosterols Formulations	Composition of Delivery Vehicle	Assessment of Oxidative Stability of Phytosterols	References
Nanoemulsion	Phosphatidylcholine Medium chain triglyceride Glycerol	Not conducted	[76]
Nanoparticles	Starch aerogel	Not conducted	[77]
Microparticles	Arabic gum, maltodextrin, polysorbate Tween 20, sodium lauryl sulphate	Not conducted	[86]
Microcapsules	Asolectin	(i) Low peroxide value (ii) POPs not screened	[87]
Micelles	Soy lecithin	Not conducted	[88]

## Data Availability

No new data were created or analyzed in this study. Data sharing is not applicable to this article.
